# Consequences of Tuning Rare-Earth RE^3+^-Site and Exchange–Correlation Energy *U* on the Optoelectronic, Mechanical, and Thermoelectronic Properties of Cubic Manganite Perovskites REMnO_3_ for Spintronics and Optoelectronics Applications

**DOI:** 10.1021/acsomega.2c01511

**Published:** 2022-08-02

**Authors:** M. Musa Saad H.-E., B. O. Alsobhi

**Affiliations:** † Department of Physics, College of Science and Arts in Al-Muthnib, Qassim University, Buraydah 52571, Saudi Arabia; ‡ Physics Department, Faculty of Science, Taibah University, Al-Madinah al-Munawarah 42353, Saudi Arabia

## Abstract

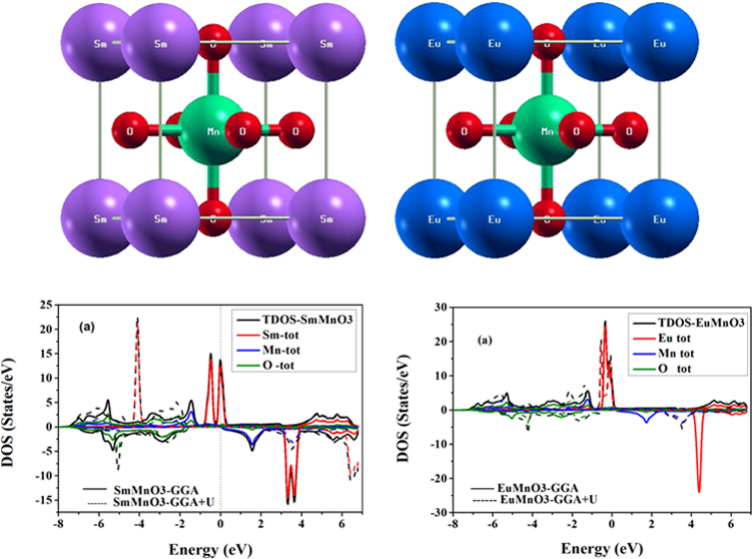

Both rare-earth SmMnO_3_ and EuMnO_3_ compounds that belong to transition-metal-based manganite perovskites REMnO_3_ have been studied deeply in this paper. The structural, elastic, optoelectronic, magnetic, mechanical, and thermoelectronic properties of cubic SmMnO_3_ and EuMnO_3_ compounds have been computed using the full-potential linearized augmented plane-wave (FP-APLW) method in the frame of density functional theory (DFT). To compute the ground-state energy, the effect of exchange–correlation potential was treated via the application of generalized gradient approximation within Perdew, Burke, and Ernzerhof (PBE-GGA) plus its corrected method (GGA + *U*). The spin-polarized results of band structures, density of states (DOS), and magnetic moments show that SmMnO_3_ and EuMnO_3_ have ferromagnetic half-metallic (FM-HM) behavior. Optical responses of dielectric function (ε(ω)) are explained by computing the real ε_1_(ω) and imaginary ε_2_(ω) parts of ε(ω), refractive index *n*(ω), extinction coefficient *k*(ω), absorption coefficient α(ω), optical conductivity σ(ω), reflectivity *R*(ω), and energy loss function *L*(ω) using GGA and GGA + *U*. Also, we computed and discussed the thermoelectronic properties of SmMnO_3_ and EuMnO_3_, including Seebeck coefficient (*S*), holes and electrons charge carrier concentration (*n*), electrical conductivity (σ/τ), power factor (*S*^2^σ/τ), figure of merit (*ZT*), thermal conductivity (κ), and specific heat capacity (*C*_V_), as a function of temperature (*T*), using GGA and GGA + *U* methods based on BoltzTrap scheme. The present results confirm the perfect mechanical and thermal stability of two perovskites which make SmMnO_3_ and EuMnO_3_ promising materials for spintronics, optoelectronics, high-temperature, and other related applications.

## 1 Introduction

Searching for efficient magnetic materials to construct spintronics and optoelectronics devices started with great enthusiasm in the last two decades by employing various theoretical and experimental techniques. Recently, huge research efforts have been directed toward studying the various physical properties of transition-metal (TM)-based perovskites and their derivative compounds. The special interest on these materials is because of their simple crystal structure, inexpensive experimental synthesis, mechanical stability, and good optoelectronic and thermoelectronic properties. Frequently, these TM-based perovskites take the common crystal structure formula ATMO_3_, where its three sites are carefully selected as: A = any metal (cation), TM = 3d, 4d, or 5d transition metal (cation), and O = oxygen (anion). As a result, this procedure has led to create various compounds of ATMO_3_, belonging to the cluster of inorganic perovskites with diverse crystal structures and gaining exclusive properties. Based on the type, ionic size, and free charges of atom that occupies A-site, five classes of ATMO_3_ compounds can be distinguished, alkali metal (A^1+^ = Li, Na, K, etc.), alkaline-earth metal (A^2+^ = Mg, Ca, Sr, etc.), rare-earth metal (A^3+^ = La, Ce, Pr, etc.), actinide metal (A^3+,4+^ = Np, Pu, Am, etc.), and general metal (A^2+^ = Sn, Pb, Bi, etc.) perovskite oxides.^1−3^ These characteristics motivated researchers to devote their attention to explore and study diverse compounds among ATMO_3_ classes in many vital fields such as solid-state physics and chemistry, materials science and engineering, and nanomaterials technology.^4,5^ The relatively simple chemical composition of ATMO_3_ materials gives them distinctive properties; they display varied structural, elastic, mechanical, magnetic, electronic, thermal, optical, and other useful properties, which make them promising materials for various modern technologies such as engineering manufactures, spintronics, optoelectronics, solar cells, fuel cells, and so on.^4−12^

Moreover, in recent years, research interest has focused on studying numerous compounds of rare-earth-based perovskite oxides that crystallize in a special formula (REMO_3_) with (RE^3+^ = lanthanide atom; M = metal). These compounds form one of the most studied classes of magnetic materials because they can deliver a broad range of functional physical and chemical properties. Therefore, several pure and doped REMO_3_ compounds have been investigated by various studies via dissimilar experimental and theoretical procedures.^11−15^ In some previous studies on REMO_3_, the RE^3+^-site was substituted by some suitable lanthanide atoms (R^3+^ = La, Ce, Pr, Nd, Sm, Eu, Gd, Dy, Lu)^7,8,15−21^ and M-site by 3d TM atoms (TM^3+^ = Cr, Fe, Mn, Co, Ni, Cu). On the other hand, there are a few studies on manganite perovskites REMnO_3_ compared to their well-known counterparts of rare-earth-based perovskite compounds. For example, Olsson et al. computed the magnetic and electronic properties of LaMnO_3_ and SmCoO_3_ in cubic structure (*Pm*3̅*m*) using first-principles density functional theory (DFT + *U*).^9^ They reported that these two perovskites can be used as cathode materials suitable for solid oxide fuel cell applications when the oxygen and cation vacancies are taken into account. By performing DFT computations, Aliabad et al. have investigated the structural, magnetic, electronic, and thermoelectric properties of GdMnO_3_ and TbMnO_3_ in orthorhombic phase.^10^ The electronic and magnetic properties of two related perovskites CeMnO_3_ and PrMnO_3_ have been computed in cubic structure (*Pm*3̅*m*).^13^ In this study, GGA + *U* computations confirmed that the two RE^3+^-based perovskites REMnO_3_ have a ferromagnetic half-metallic (FM-HM) nature in both cases of (RE^3+^ = Ce^3+^) and (RE^3+^ = Pr^3+^). REMnO_3_ with (RE^3+^ = Sm^3+^)^20^ and (RE^3+^ = Eu^3+^)^21^ show orthorhombic (*Pbnm*) structure at high temperatures.

Motivated by the paucity of studies on these materials, this paper presents a systematical study on two related compounds of rare-earth-based manganite perovskites REMnO_3_, where RE^3+^ site is selected as (RE^3+^ = Sm, Eu). Also, further investigations on their optoelectronic, mechanical, and thermoelectronic properties are performed using first-principles methods. It is interesting to note that the special electron configuration of Mn atom (Mn: 3d^5^ 4s^2^) in REMnO_3_ yields unique physical properties of these two compounds. All of the investigations are carried out using the first-principles DFT computations under the generalized gradient approximation (GGA) plus exchange–correlation (XC) method (GGA + *U*). Besides, this study devotes to expose the consequences of substituting RE^3+^ site and application of the GGA + *U* technique on these properties. We believe that this study will be an important addition to the existent data of magnetic perovskite materials, as its results provide detailed and useful information for RETMO_3_ compounds. Following this Section 1, the rest of this paper is divided as follows: In Section 2, a brief description of the method and computations details is given. Section 3 is devoted to displaying the details of GGA and GGA + *U* results and discussing their outputted properties for SmMnO_3_ and EuMnO_3_, and finally, the main summary points and conclusions of this study are summarized in Section 4.

## 2 Method of Computations

First, for both cubic manganite perovskites, SmMnO_3_ and EuMnO_3_, with (*a* = *b* = *c*) and (α = β = γ = 90°), exhibiting space group number 221 (*Pm*3̅*m*), as displayed in Figure S1, are specified by considering the atomic sites and positions in their primitive unit cell as follow: Sm^3+^/Eu^3+^ at 1a (0, 0, 0), Mn^3+^ at 1b (1/2, 1/2, 1/2), and O^2–^ at 3c (1/2, 1/2, 0), (1/2, 0, 1/2) and (0, 1/2, 1/2). Second, to achieve their ground-state energy and all equilibrium structural parameters, the structural optimizations are performed using the WIEN2k,^22^ where the total energy (*E*) is computed and designed against varying the unit cell volume for these compounds. WIEN2k computations are based on the all-electron self-consistent full-potential linearized augmented plane-wave (FP-APLW) method in the frame of DFT.^23^ Also, the effect of exchange and correlation (XC) between the electrons is treated using the GGA approximation^24^ executed via operating the Perdew, Burke, and Ernzerhof (PBE) method.^25^ Moreover, the spin-polarized GGA + *U* functional is implemented to include the XC effect of 4f–4f and 3d–3d electrons in SmMnO_3_ and EuMnO_3_ by incorporating the on-site Coulomb interaction energy (*U*) and Hund’s rule exchange energy (*J*)
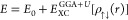
1where the Hubbard energy is described as
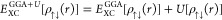
2Here, *U*[ρ_↑↓_(*r*)] represents the energy of Hubbard functional plus double-counting term *E*^Hub^[{*n*_mm_^*l*σ^}] – *E*^*d*c^[*n*^*l*σ^] for the localized 3d and 4f orbitals with occupation number *m* and spin σ. Also, the energy in (eq 2) can be defined via
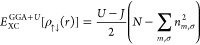
3

In WIEN2k, the Hubbard parameters were set as optimized energies, (*U*_eff_ = 7.0 eV) for RE^3+^-4f states and (*U*_eff_ = 5.0 eV) for Mn^3+^-3d states,^9,10,26^ in which the effective Hubbard energy (*U*_eff_) is related to *U* and *J* energies by
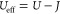
4

Third, for elucidating the consequences of RE^3+^ site and *U* on structural, elastic, thermal, electronic, magnetic, thermoelectronic, and optical properties, as well as total density of states, partial density of states and band structures as a function of the unit cell energy are methodically computed using the GGA + *U* method.

To ensure correct convergence for all computations, the cutoff energy, separation energy between valence and core states, of (*E*_cutoff_ = −6.5 Ry; ∼ −95.2399 eV), number of *k*-points of (*k*-points = 2000), and a mesh of (12 × 12 × 12) *k*-points in their first Brillouin zone (BZ) were set. Nonmagnetic (NM), antiferromagnetic (AFM), and spin-polarized ferromagnetic (FM) phases were selected to examine the GGA equilibrium structural parameters of two compounds. Also, the joined plane-wave (PLW) parameter, which includes the smallest radius of muffin-tin sphere (*R*_MT_ = 2.50 (RE^3+^), 1.80 (Mn^3+^), and 1.60 (O^2–^) au), and the largest reciprocal lattice vector for the expansion of flat wave function (*K*_max_)^27,28^ was set as (*R*_MT_*K*_max_ = 8.0). Setting *R*_MT_ at these values ensures that there is no charge escape from the inner atomic core of RE^3+^, Mn^3+^, and O^2–^ sites, besides achieving an accurate for their energy eigenvalues convergency. Hence, the potential *V*(*r*) and charge density ρ(*r*) within these MTs are expanded in terms of crystalline spherical harmonics up to the value of angular momenta (*L*_max_ = 10.0) and the PLW expansion has been applied on the interstitial region sites. Moreover, the Fourier expansion parameter was set as (*G*_max_ = 18.0) to delimit the magnitude of the largest vector in ρ(*r*). The values of energy and charge convergence were chosen as (*E*_Conv._ = 0.00001 Ry) and (ρ_Conv._ = 0.0001e), respectively, during the self-consistency computational cycles of GGA for two compounds. The elastic properties were computed by exploiting the Charpin elastic code.^29^ The thermoelectric properties, Seebeck coefficient (*S*), hole and electron charge carrier concentration (*n*), electrical conductivity (σ/τ), power factor (*S*^2^σ/τ), figure of merit (*ZT*), thermal conductivity (κ), and specific heat capacity (*C*_V_) of SmMnO_3_ and EuMnO_3_ are computed using the Boltzmann-transport-dependent code (BoltzTrap).^30,31^ The values of σ and κ are dependent on the relaxation time (τ), which was taken as a constant and set as (τ = 10^–14^ s) inside the BoltzTrap code, while the value of *S* is independent.^31,32^

## 3 Results and Discussion

Here in this section, we report the details of computed properties for the two concerned REMnO_3_ compounds developed in this study along with the analysis and discussion of their attained results for showing the applicable conclusions of our study.

### 3.1 Structural Properties

**Figure 1:**
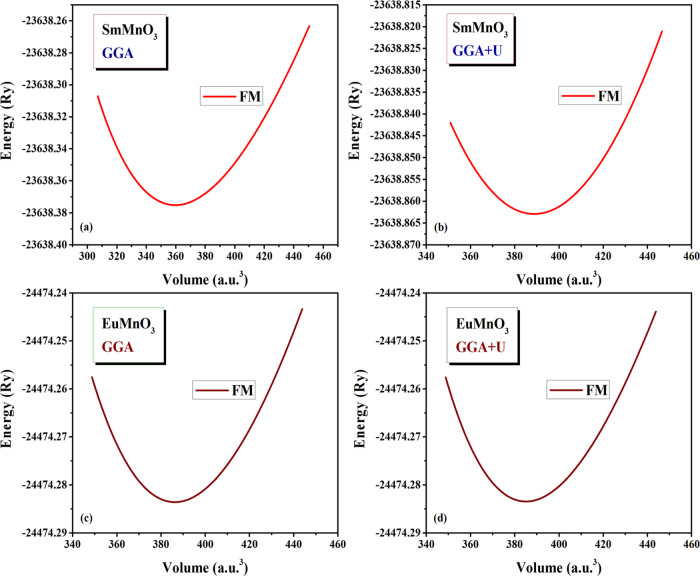
Structural optimization of perovskites (a, b) SmMnO_3_ and (c, d) EuMnO_3_ in the FM state using GGA and GGA + *U* methods.

First, the lattice parameters of the cubic (*Pm*3̅*m*) unit cell for REMnO_3_ are fixed at (*a* = 3.9800 Å) and their crystal volumes are built by setting the general atomic positions: RE^3+^ at (0, 0, 0), Mn^3+^ at (1/2, 1/2, 1/2), and O^2–^ at (1/2, 1/2, 0), (1/2, 0, 1/2) and (0, 1/2, 1/2), (Figure S1). Next, to perform systematic structural optimizations, at room temperature, we have computed the total energy (*E*) per unit cell volume (*V*) of the nonmagnetic (NM), antiferromagnetic (AFM), and ferromagnetic (FM) states. The plots of computed *E* vs *V* for the stable FM state of SmMnO_3_ and EuMnO_3_ compounds are presented in Figure 1. Figures S2–S5 show the three plots of NM, FM, and AFM states for these two compounds. All of these *E* vs changed *V* are fitted to Murnaghan’s equation of state (MEOS)^33^
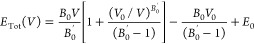
5

This allows evaluating the main ground-state structures of two perovskites, including ground total energy (*E*_0_), equilibrium volume (*V*_0_), lattice constant (*a*_0_), main interatomic bond distances (RE–O, Mn–O), bulk modulus (*B*_0_), and its first pressure derivative value (*B*_0_′).

From these plots, it is found that the FM state (Figure 1a–d) is the most favorable in total energy with regard to the corresponding NM and AFM states (see Figures S2–S5), confirming that FM is the stable ground state of the cubic SmMnO_3_ and EuMnO_3_ compounds. The computed structural properties of the stable state of SmMnO_3_ and EuMnO_3_ are listed in (Table 1). Table S1 contains additional results of the computed structural properties of the two compounds in their NM, FM, and AFM states. Through comparing the optimized results in Table 1, it can be concluded that the results of *a*_0_, *V*_0_, and bond distances of SmMnO_3_ seem to be larger than that of EuMnO_3_; however, the values of *B*_0_ and *B*_0_′ for EuMnO_3_ are larger than the other compound. These variations indicate that SmMnO_3_ is less compressible than EuMnO_3_. The obtained structural properties are consistent with the previous predictions for similar REMnO_3_ perovskites computed via utilizing the GGA and GGA + *U* methods within WIEN2k code.^8,9,13,34−38^

**Table 1:** Computed Structural Properties of Stable FM State (Figure 1) of Perovskites SmMnO_3_ and EuMnO_3_

REMnO_3_	SmMnO_3_		EuMnO_3_	
parameter/method	GGA	GGA + *U*	GGA	GGA + *U*
lattice constant *a*_0_ (Å)	3.8520	3.8623	3.8539	3.8500
equilibrium volume *V*_0_ (Å^3^)	57.154	57.617	57.239	57.068
bulk modulus *B*_0_ (GPa)	165.75	156.22	175.03	176.38
first pressure derivative *B*_0_′	3.7685	4.8572	4.2987	5.7444
ground total energy *E*_0_ (Ry)	–23638.862296	–23638.862917	–24474.283599	–24474.283468
bond distance RE–O (Å)	2.8855	2.8431	2.8372	2.8372
bond distance Mn–O (Å)	2.0404	2.0104	2.0062	2.0062
bond distance RE–Mn (Å)	3.5340	3.4821	3.4749	3.4749

### 3.2 Elastic Properties

To describe the mechanical properties of cubic perovskite compounds SmMnO_3_ and EuMnO_3_, the three basic elastic constants (*C_ij_* = *C*_11_, *C*_12_, *C*_44_), besides their derived mechanical constants, are computed and tabulated in Table 2. Essentially, these elastic parameters provide information about the impacts of applying force on crystal structures. By evaluating the elastic constants, we can easily determine their mechanical responses like hardness, rigidity, brittleness or ductility, and stability. Based on the utilization of Charpin elastic code embedded in WIEN2k, the stability of cubic perovskites SmMnO_3_ and EuMnO_3_ can be confirmed via the Born elastic stability criteria^38^
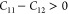
6
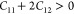
7
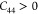
8
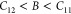
9

**Table 2:** Computed Elastic Properties of Perovskites SmMnO_3_ and EuMnO_3_

	SmMnO_3_		EuMnO_3_	
elastic parameters	GGA	GGA + *U*	GGA	GGA + *U*
elastic constants (*C_ij_*; *i* = *j* = 1); *C*_11_ (GPa)	240.66	365.581	257.945	358.03
elastic constants (*C_ij_*; *i* = 1, *j* = 2); *C*_12_ (GPa)	182.69	125.068	158.748	144.37
elastic constants (*C_ij_*; *i* = *j* = 4); *C*_44_ (GPa)	73.677	80.498	89.589	72.582
elastic anisotropy factor; *A*	2.5419	0.669383	1.80628	0.6794
fitted Hill bulk modulus; *B* (GPa)	201.72	205.101	191.164	215.90
Hill bulk modulus from *C_ij_*; B (GPa)	202.02	205.239	191.814	215.59
Hill shear modulus; *G* (GPa)	50.685	94.5834	70.6675	84.769
Young modulus; *E* (GPa)	140.31	245.966	188.815	224.84
Cauchy’s pressure; *C*″(GPa)	109.02	44.57	69.159	71.791
Pugh’s ratio; K	3.9857	2.16992	2.71431	2.5433
Poisson’s ratio; υ	0.3442	0.30026	0.335939	0.3262
Vickers hardness; *H*_V_ (GPa)	61.084	251.676	138.046	180.69

From elastic data shown in Table 2, it is clear that the values of *C*_11_, *C*_12_, and *C*_44_ obey Born’s stability, which confirms that SmMnO_3_ and EuMnO_3_ are mechanically stable. Also, the crystal structures of two perovskites possibly experience the pure shear deformation as a response to uniaxial compression since both have (*C*_11_ > *C*_44_) by about 70%.^31^ Moreover, *C*_44_ (SmMnO_3_) > *C*_44_ (EuMnO_3_) indicates that SmMnO_3_ perovskite possesses high resistance against this shear deformation along the [100] plane, making it a more stiff material. By means of *C_ij_* values, other mechanical constants including elastic anisotropy factor (*A*), Hill bulk modulus (*B*), Hill shear modulus (*G*), Young’s modulus (*E*), Cauchy’s pressure, Vickers hardness (*H*_V_), and Poisson’s ratio (υ) can be computed via the following equations^31,40,41^
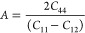
10
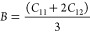
11
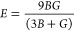
12
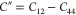
13
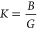
14
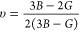
15
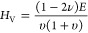
16

Here, *G* denotes the computed value of Hill shear, which refers to the average value of Reuss and Voigt shears, and is used to designate the response of the crystal to the shearing strain.^42,43^ According to the results of the above elastic parameters (Table 2), the mechanical properties of the studied perovskites are analyzed as follows. It is clearly seen that the two crystals are completely anisotropic; SmMnO_3_ shows the highest value (*A* > 1.0) compared to EuMnO_3_ that gives *A* < 1.0. The values of *B* confirm that the crystal structures of two perovskites have significant hardness, which measures the resistance against changing their geometric shape or evaluates their resistance to the fracture. The fitting and computed values are nearly equal, where EuMnO_3_ has B greater than SmMnO_3_, and there is strong agreement between the *B* from MEOS (Table 1) with the *B* obtained via elastic constants method (Table 2). Also, it is well known that the *E* value determines the stiffness of the crystal. So, we find that the two perovskites obey this condition, as the first compound SmMnO_3_ has a greater stiffness than EuMnO_3_.

Another important elastic factors include Pugh’s ratio (*B*/*G*), Cauchy’s pressure, and Poisson’s ratio (υ) that are computed to decide the ductile and brittle nature of the crystal structures.^23^ (*C*″ > 0) reflects ductile compounds, as in our perovskites (*C*″ = 109.02) and (*C*″ = 71.791), whereas (*C*″ < 0) indicates a brittle feature. SmMnO_3_ and EuMnO_3_ give *K* = 3.99 and 2.54, respectively, more than the critical value (*K* = 1.75), which means that the two compounds are ductile. The value of υ is an important indicator about the bonding forces in crystal structures; frequently, the lower and upper limits of υ for the central forces in crystals are (υ = 0.25) and (υ = 0.50), respectively. Metal crystals show (υ = 0.25–0.45) with a very few exceptions.^31^ The computed values of υ are about (υ = 0.34) and (υ = 0.33) for SmMnO_3_ and EuMnO_3_, respectively. These indicate that they comprise a metallic bonding and the interatomic forces between their atoms are central forces. Moreover, the brittle crystal structures show (υ < 0.26); otherwise, they are considered ductile materials.^42^ Accordingly, the present results confirm that the studied perovskites are ductile materials. Furthermore, the values of *H*_V_ parameter, which confirm the ability of crystals to resist denting, are computed under ambient conditions. SmMnO_3_ and EuMnO_3_ give largest values (*H*_V_ = 61.1 GPa) and (*H*_V_ = 180.7 GPa), respectively, indicating high hardness of these crystal structures. The small differences between GGA and GGA + *U* values of elastic constants are due to the effect of *U* energy on the structural parameters of SmMnO_3_ and EuMnO_3_ (Table 1), where we found that the computed lattice constants by GGA + *U* are greater than those obtained using the GGA method.

### 3.3 Thermal Properties

The thermodynamic properties of two perovskites SmMnO_3_ and EuMnO_3_, which depend on their elastic properties, can be predicted by computing the thermal parameters like melting temperature (*T*_m_), average wave velocity (*v*_m_), and Debye temperature (Θ_D_). The value of *T*_m_ for these crystals is computed by means of their corresponding elastic constant *C*_11_ using the simple formula^43,44^
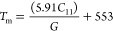
17

The average wave velocity *v*_m_ is computed from^32,43^
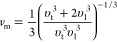
18Here, *v*_t_ and *v*_l_ represent the transverse and longitudinal components of the average sound velocity, respectively, that can be acquired using the values of the density (ρ), *G*, and *B*, as follows^44^
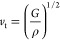
19
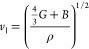
20

The value of basic parameter Θ_D_ is determined using the value of *v*_m_ in eq 18 and other physical constants via the equation^32,44^
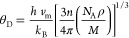
21where *h* is Plank’s constant, *k*_B_ is the Boltzmann constant, *N*_A_ is Avogadro’s number, *M* is the molecular weight, and *n* is the number of atoms in the unit cell. It can be seen that Θ_D_ correlates to fundamental physical properties of solid crystals, such as crystal structure, elastic constants, melting temperature, specific heat, enthalpy, thermal conductivity, thermal loss, and thermal expansion.

Based on the above formulas (eqs 17–21), the computed results of thermal parameters, *T*_m_, *v*_t_, *v*_l_, *v*_m_, and θ_D_, for perovskites SmMnO_3_ and EuMnO_3_, are displayed in Table 3. From these results, it is clearly seen that the computed values of thermal parameters (Table 3) are mainly dependent on the obtained values of elastic constants of the studied perovskite crystals SmMnO_3_ and EuMnO_3_ (Table 2). Therefore, if the crystals have a large elastic constant *C*_11_, as for our perovskite, they will show higher values of average sound velocity.^32,45^ According to the present results, the high obtained values of Debye temperature (θ_D_ = 305–317 K) and melting temperature (*T*_m_ = 2344–2373 K), which is accompanied by other preferred properties like specific heat, enthalpy, and thermal conductivity, besides their mechanical stability, we expect that SmMnO_3_ and EuMnO_3_ materials may be promising candidates for many applications in the high-temperature technology.

**Table 3:** Computed Thermal Properties of Perovskites SmMnO_3_ and EuMnO_3_

	SmMnO_3_		EuMnO_3_	
thermal parameters	GGA	GGA + *U*	GGA	GGA + *U*
melting temperature; *T*_m_ (K) ± 300	1975.3	2713.6	2077.5	2668.9
transverse velocity; *ν*_t_ (m/s)	1267.3	1729.1	1487.2	1630.1
longitudinal velocity; *ν*_ι_ (m/s)	2922.9	3236.1	2992.1	3209.5
average wave velocity; *ν*_m_ (m/s)	1431.6	1931.3	1668.9	1826.9
debye temperature; θ_D_ (K)	259.54	350.46	303.21	331.73

### 3.4 Magnetic Properties

The computed values of partial and total (*M*_Total_) spin magnetic moments per unit cell of the two perovskites SmMnO_3_ and EuMnO_3_, using GGA and GGA + *U* methods, are summarized in Table 4. The main remark from these results is that the GGA + *U* enhances the partial spin magnetic moment on Mn^3+^ ions (*M*_Mn_), which causes an increase in the corresponding *M*_Total_ in two compounds. Also, it can be seen the major contribution to *M*_Total_ is due to the spin magnetic moments of Sm^3+^/Eu^3+^ ions (*M*_Sm/Eu_) and *M*_Mn_, whereas the interstitial sites (*M*_Int_) and O^2–^ ions (*M*_O_) have negligible contribution. The large exchange splitting between the spin-down and spin-up partial states in Sm^3+^/Eu^3+^-4f and Mn^3+^-3d orbitals has the highest contribution in the *M*_Total_ of SmMnO_3_ and EuMnO_3_. The obtained results indicate the presence of half-metallic ferromagnetic (HM-FM) properties in two perovskites SmMnO_3_ and EuMnO_3_. GGA shows that the value of *M*_Total_ for these perovskites is 8.036μ_B_ and 9.996μ_B_, respectively. When we applied the Hubbard energy *U* using GGA + *U* method, *M*_Total_ increased significantly to 9.000μ_B_ and 10.01μ_B_, respectively. The computed *M*_Mn_ in two compounds is in agreement with the theoretical spin magnetic moment, where according to Hund’s theory and due to the existence of crystal field, the spin occupation of partial orbitals of Mn^3+^ in perovskites SmMnO_3_ and EuMnO_3_ takes the form Mn^3+^-3d^4^: t_2g_^3↑^ t_2g_^0↓^ e_g_^1↑^ e_g_^0↓^; (*M*_S_ = 2μ_B_). Accordingly, the FM in these two compounds is directed by the exchange interaction between the 3d and 2p electrons through the long-range path Mn^3+^ (3d^4^)–O^2–^ (2p)–Mn^4+^ (3d^3^). Due to this interaction, the valence electron in 3d-e_g_ tends to make a real hopping between the orbitals, from Mn^3+^ to O^2–^ to Mn^4+^ in parallel spins alignment, which yields FM-stable configurations Mn^4+^-3d^3^: t_2g_^3↑^ t_2g_^0↓^ e_g_^0↑^ e_g_^0↓^ and Mn^3+^-3d^4^: t_2g_^3↑^ t_2g_^0↓^ e_g_^1↑^ e_g_^0↓^. The values of *M*_O_ are very little, and the opposite signs of *M*_O_ and *M*_Mn_ via GGA + *U* reveal the antiparallel alignment of electron spins in 3d and 2p orbitals. As a result of the application of *U* energy within GGA + *U*, the on-site Coulomb interaction between 3d–3d electrons through 2p states lowers the energy of the occupied 3d orbitals and increase the energy of the unoccupied 3d orbitals in SmMnO_3_ and EuMnO_3_. Furthermore, this interaction enhances the localization of related 3d orbitals and the local spin magnetic moments in Mn^3+^ ions.

**Table 4:** Computed Magnetic Properties of Perovskites SmMnO_3_ and EuMnO_3_

REMnO_3_	SmMnO_3_		EuMnO_3_	
moment/method	GGA	GGA + *U*	GGA	GGA + *U*
magnetic moment on interstitial; *M*_Int_ (μ_B_)	0.3314	0.4083	0.4503	0.4572
magnetic moment on RE^3+^; *M*_Sm/Eu_ (μ_B_)	5.3992	5.4023	6.5073	6.508
magnetic moment on Mn^3+^; *M*_Mn_ (μ_B_)	2.1629	2.3994	3.0432	3.0482
magnetic moment on O^2–^; *M*_O_ (μ_B_)	0.0475	–0.0700	0.0016	–0.0021
magnetic moment on REMnO_3_; *M*_Total_ (μ_B_)	8.0361	9.0000	9.9961	10.007

### 3.5 Electronic Properties

**Figure 2:**
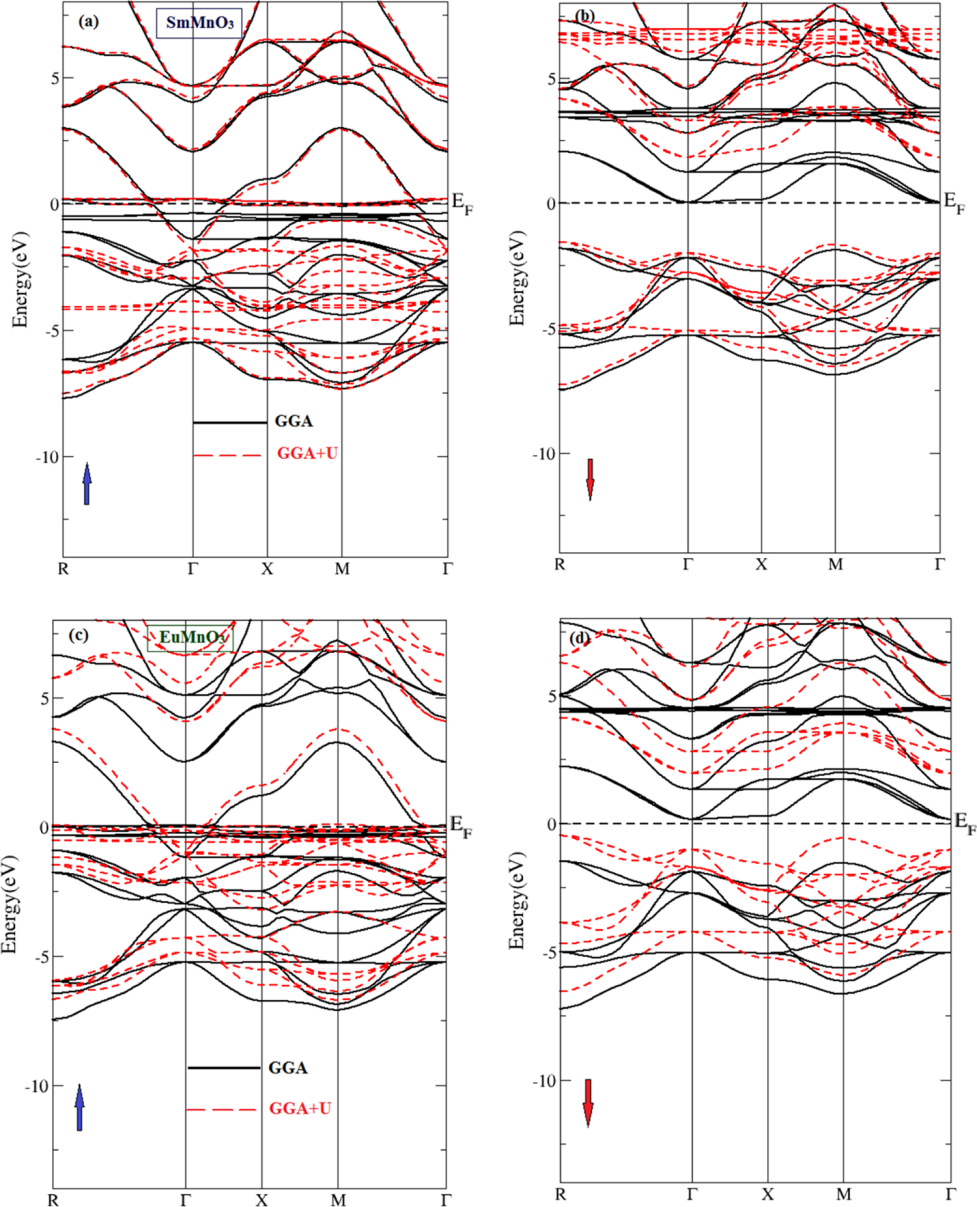
Computed spin-up (↑) and spin-down (↓) band structures per unit cell of perovskites (a, b) SmMnO_3_ and (c, d) EuMnO_3_ using GGA and GGA + *U* methods. The horizontal line at *E* = 0.0 eV represents the Fermi level (*E*_F_).

The main results of spin-polarized distribution of band structures in SmMnO_3_ and EuMnO_3_ perovskites at their optimized lattice parameters are computed using the GGA and GGA + *U* methods and plotted in Figure 2a–c, respectively, along their high-symmetry *k*-points in the first Brillion zone. It is evident from all of these plots that there are some bands that cross the Fermi level (*E*_F_) in spin-up states and make a band gap (*E*_g_) in spin-down states within GGA and GGA + *U* (Table 5). This indicates that the two perovskites SmMnO_3_ and EuMnO_3_ have an electronic half-metallic (HM) nature. Compared to GGA, we find that the introduction of Hubbard energy *U* enlarges the spin-down *E*_g_ of two perovskites, which refers to the major effect of repulsion energy within the GGA + *U* treatment.

**Table 5:** Computed Electronic Properties of Perovskites SmMnO_3_ and EuMnO_3_

REMnO_3_	SmMnO_3_		EuMnO_3_	
*E*_g_/method	GGA	GGA + *U*	GGA	GGA + *U*
energy gap in spin-up *E*_g_↑ (eV)	0.0000	0.0000	0.0000	0.0000
energy gap in spin-down *E*_g_↓ (eV)	1.9150	3.4190	1.6400	2.4460

**Figure 3:**
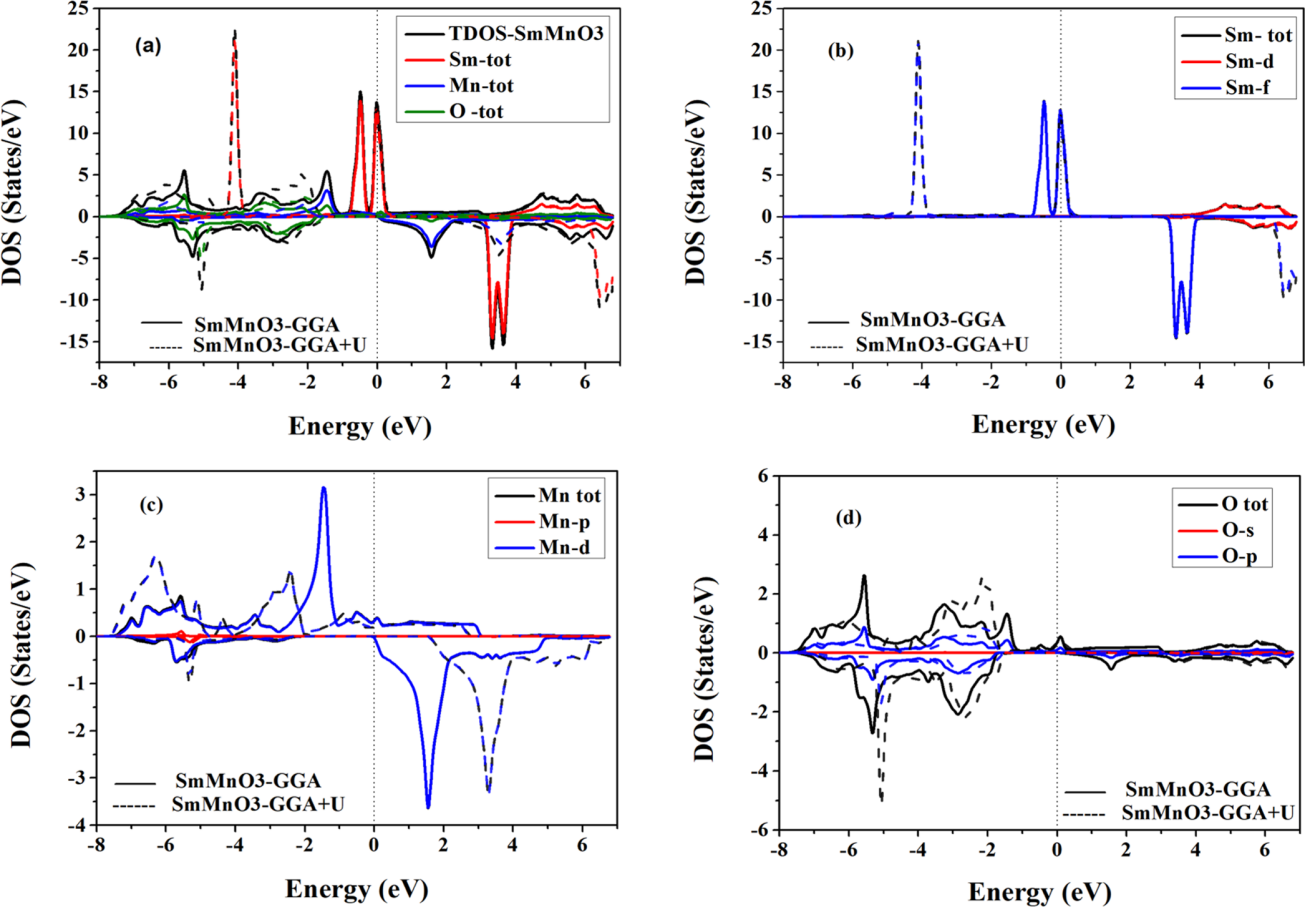
Computed spin-up and spin-down (a) total and (b–d) partial densities of states per unit cell of perovskite SmMnO_3_ using GGA and GGA + *U*. The vertical dashed line at (*E* = 0.0 eV) represents the Fermi level (*E*_F_).

**Figure 4:**
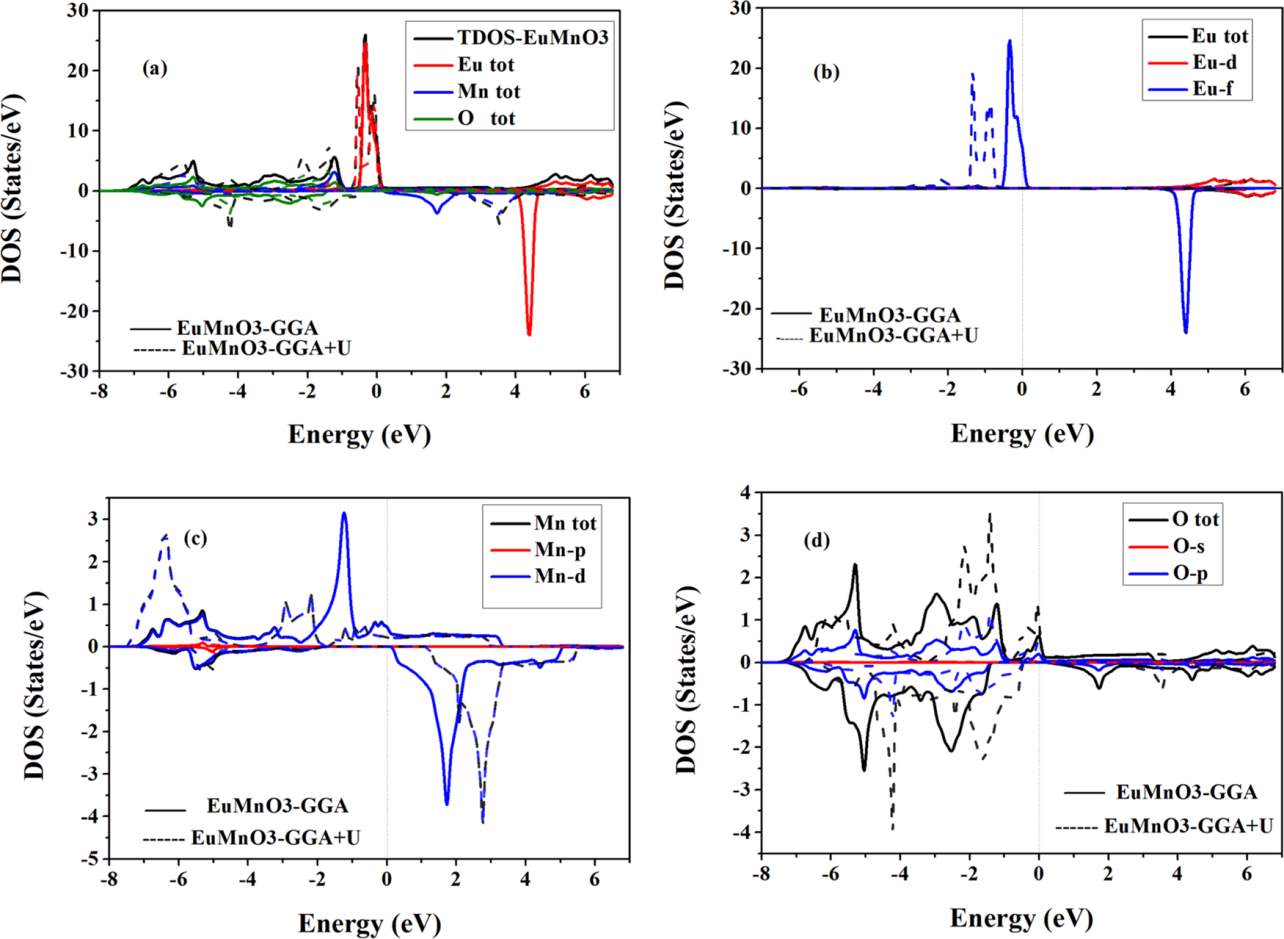
Computed spin-up and spin-down (a) total and (b–d) partial densities of states per unit cell of perovskite EuMnO_3_ using GGA and GGA + *U*. The vertical dashed line at (*E* = 0.0 eV) represents the Fermi level (*E*_F_).

Figures 3 and 4 show the plots of total density of states (TDOS) and partial density of states (PDOS) as a function of unit cell energy for two perovskite compounds SmMnO_3_ and EuMnO_3_, respectively, computed using GGA and GGA + *U*. Besides, to explain the different contributions that gave the HM nature in the obtained band structures and TDOSs, we have also computed and plotted the PDOSs per atom for the energetic states Sm^3+^/Eu^3+^ (4d, 4f), Mn^3+^ (3p, 3d), and O^2–^ (2s, 2p). First, it can be clearly seen from the TDOS of SmMnO_3_ (Figure 3) and EuMnO_3_ (Figure 4) that there is an energy gap (*E*_g_) in spin-down TDOSs for these two perovskite compounds, which confirms their HM nature. Also, SmMnO_3_ shows larger values of *E*_g_ than those for EuMnO_3_ within GGA and GGA + *U* (Table 5). From TDOSs (Figures 3a and 4a), it can be noted that the overlapping of the conduction states through the *E*_F_, namely, the bandwidth of HM in spin-up panel, increases from SmMnO_3_ to EuMnO_3_, which indicates the effect of exchange–correlation energy *U* plus the additional electron in Eu-4f^7^ orbitals than in Sm-4f^6^ ones.

Furthermore, the effect of projected PDOSs of three atoms Sm/Eu (Figures 3b and 4b), Mn (Figures 3c and 4c), and O (Figures 3d and 4d) on the TDOSs of their corresponding compounds SmMnO_3_ and EuMnO_3_ indicates that the band structures and TDOSs can be divided into three main regions. In the first region, −6.0 to −2.0 eV, we find that the orbital contribution comes mainly from Mn-3d plus O-2p electrons to form the valence bands of these compounds. The second region, which covers the conduction band from −2.0 to +2.0 eV, represents the orbital hybridization contributed by the spin-up states of Sm/Eu-4f, Mn-3d plus O-2p. Here, there is an exchange splitting between the spin-down and spin-up partial states of Sm/Eu-4f and Mn-3d orbitals, which contribute the majority part of the total spin magnetic moments of the unit cell of SmMnO_3_ and EuMnO_3_ compounds (Table 4). However, the third region, +2.0 to +6.0 eV, shows the contribution of Sm/Eu-4d and Mn-3d states plus a small amount coming from O-2p. The obtained TDOSs indicate that perovskites exhibit an HM nature through GGA and GGA + *U* methods with little difference in the value of *E*_g_, where the exchange–correlation energy *U* opens the *E*_g_ in TDOSs of both SmMnO_3_ and EuMnO_3_ and this induces these perovskites to produce HM with an *E*_g_ in spin-down panel (Table 5). This enhancement in band structures, TDOSs, and PDOSs is in conformity with the major trends detected by some previous studies on rare-earth perovskites by employing GGA and GGA + *U* computations^8,13,34,38^

The electronic charge density distributions give a clear picture of the nature of chemical bonds in the crystal structures. In Figure S6a,b, the computed charge density per unit cell of SmMnO_3_ and EuMnO_3_ are presented using the GGA and GGA + *U* methods. The exchange–correlation energy *U* has a weak effect on electronic charge density. The positions and number of contour lines in these charge density illustrations confirm the distributions of the partial and total charge densities. The shape of contour lines distributions around the cations Sm^3+^/Eu^3+^ and anions O^2–^ is obviously spherical, which confirms the strong ionic nature of Sm^3+^/Eu^3+^–O^2–^ bonds. Due to the large electronegativity difference between Sm^3+^/Eu^3+^ and O^2–^, their energetic charges transfer from the cations Sm and Eu to the anions O^2–^, while the dense of charge density around the middle cations Mn^3+^ and anions O^2–^ is regularly distributed, which confirms the covalent bonding character between Mn^3+^–O^2–^ in their octahedra MnO_6_ through the long-range −Mn^3+^–O^2–^–Mn^4+^–. This nature is due to the 2p–3d hybridizations of cations Mn^3+^-3d and anions O^2–^-2p electrons near the *E*_F_, which can be visibly observed in Figure S2. Therefore, two mixed types of chemical bonds, i.e., ionic and covalent bonds, are predicted to govern the electronic and magnetic structures of two perovskites crystals SmMnO_3_ and EuMnO_3_, in agreement with that expected for related REMnO_3_ perovskites.^8,13,38^

### 3.6 Thermoelectric Properties

**Figure 5:**
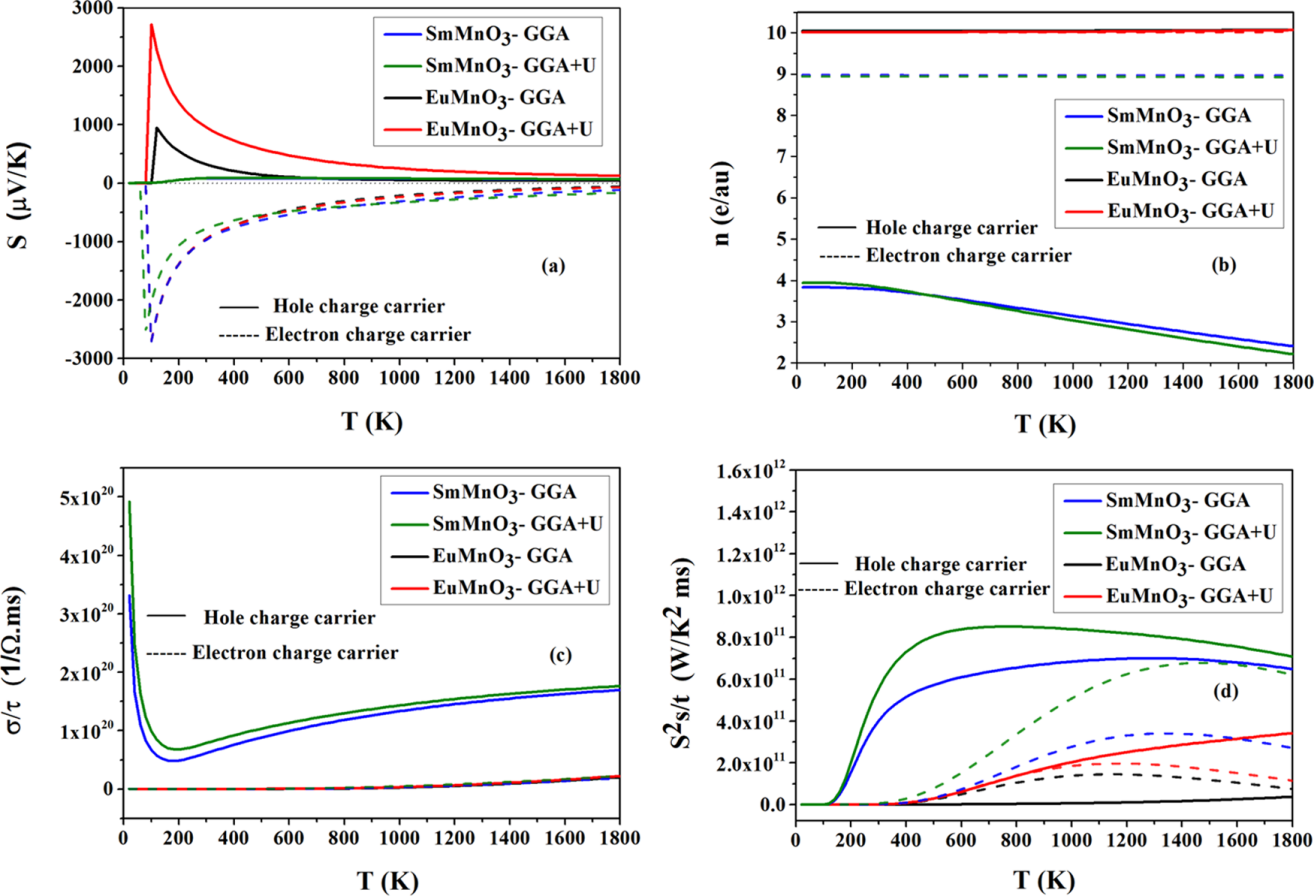
Variation of computed (a) Seebeck coefficient (*S*), (b) charge carrier concentration (*n*), (c) electrical conductivity (σ/τ), and (d) power factor (*S*^2^σ/τ) with temperature (*T*) for charge carriers, holes (solid line), and electrons (dash line) of perovskites SmMnO_3_ and EuMnO_3_ using GGA and GGA + *U*.

**Figure 6:**
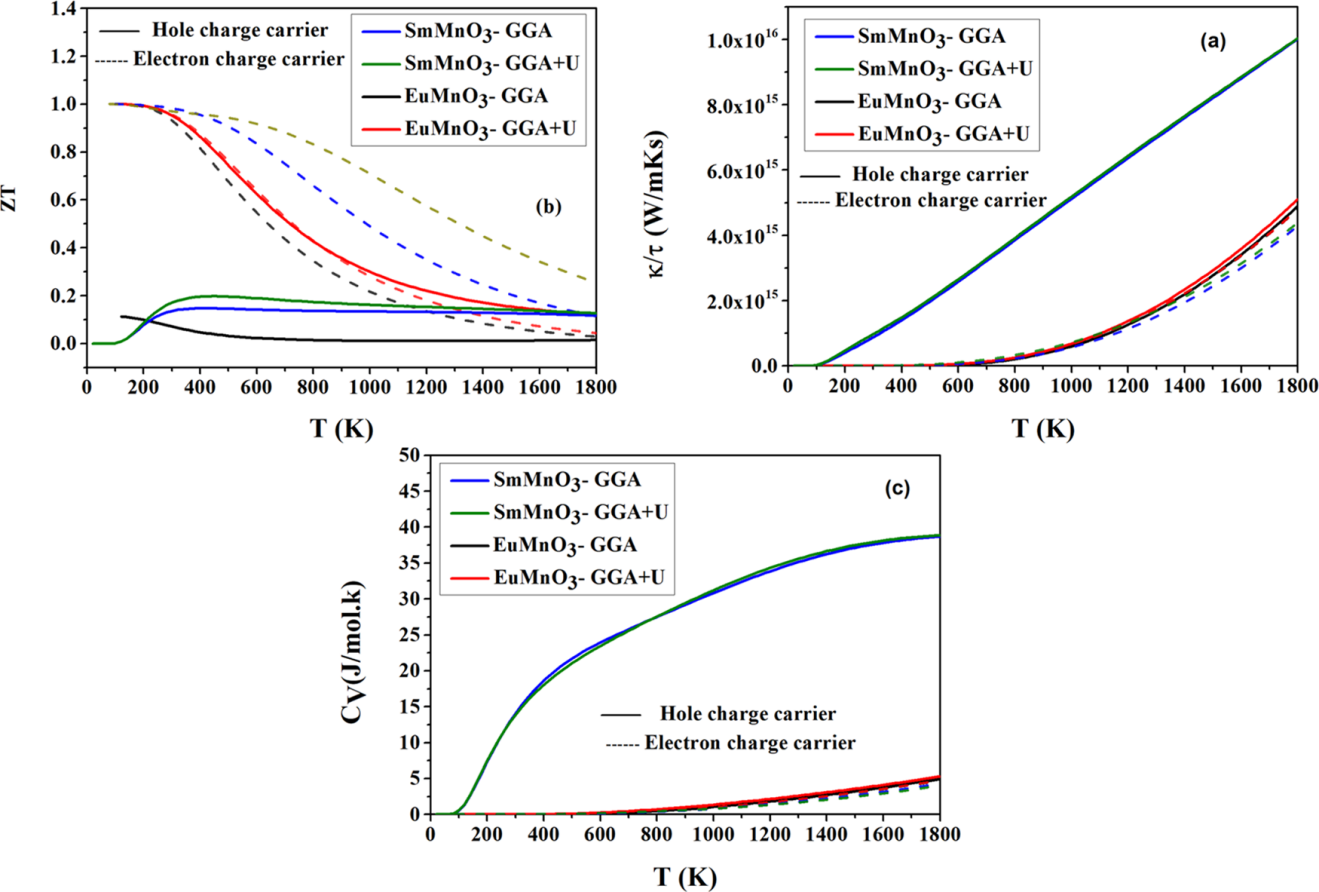
Variations of computed (a) figure of merit (*ZT*), (b) thermal conductivity (κ), and (c) specific heat capacity (*C*_V_) with temperature (*T*) for charge carriers, holes (solid line), and electrons (dash line) of perovskites SmMnO_3_ and EuMnO_3_ using GGA and GGA + *U*.

The thermoelectric properties of two perovskites SmMnO_3_ and EuMnO_3_, which evaluate their ability to convert thermal energy directly to electrical energy, and verse versa, are computed using the BoltzTrap theory. Figures 5 and 6 illustrate the results of computed thermoelectric properties as a function of temperature in the range (*T* = 0–1800 K), under the constant relaxation time approximation of the charge carriers, for SmMnO_3_ and EuMnO_3_, using GGA and GGA + *U* methods. From Figure 5a, which illustrates the variation of Seebeck coefficient (*S*) of two perovskite compounds within the *T*, it can be seen that the maximum absolute value (*S*_max_) related to electron doping is larger than that for hole doping. This shows that the majority of charge carriers for the conduction in two compounds are electrons rather than holes. Table 6 summarizes the computed values of *S*_max_ with their corresponding *T* and the charge carrier concentration (*n*) for electron doping and hole doping. It is seen from these data that the values of *n* are positive for *S*_max_ values of electron doping, which assumes that the two perovskites possess p-type doping characteristics. However, GGA + *U* gives equivalent values (*S*_max_ = −2720 μν/K) for EuMnO_3_, indicating that the conduction occurs through both electrons and holes. In Figure 5b, we show the computed results of variation of *n* vs *T*, which is mostly linear within GGA and GGA + *U* methods and proportional with *T*. As *T* increases, the thermal excitations of two compounds get high, which increases the value of *n*, and this causes an increase in the number of free electrons that move from valence bands through *E*_F_ to conduction bands and generates hole–electron pairs in crystals.

**Table 6:** Computed Thermoelectric Properties of Perovskites SmMnO_3_ and EuMnO_3_

		SmMnO_3_		EuMnO_3_	
charge carriers	thermoelectric parameters	GGA	GGA + *U*	GGA	GGA + *U*
electron doping	maximum Seebeck coefficient; *S*_max_ (μν/K)	–2720	–2550	–2720	–2720
	corresponding temperature; *T* (K)	100	80	100	100
	carrier concentration; *n* (e/au)	8.9762	8.9427	10.049	10.0145
	thermoelectric figure of merit; *ZT*	0.9999	0.9999	0.9999	0.9999
hole doping	maximum Seebeck coefficient; *S*_max_ (μν/K)	82.6	89.1	951	2720
	corresponding temperature; *T* (K)	360	420	120	100
	carrier concentration; *n* (e/au)	3.7393	3.7116	10.0491	10.0145
	thermoelectric figure of merit; *ZT*	0.1458	0.9999	0.1123	0.9999

Figure 5c shows the computed electrical conductivity relative to the relaxation time (σ/τ) of hole and electron charge carriers with the variation of *T*. SmMnO_3_ and EuMnO_3_ show similar patterns of total electrical conductivity. The computed values of σ/τ increase directly with *T*, and inversely with *S*, which is consistent with the Mott formula of thermoelectric for metal.^32,42,46^ This feature is in agreement with the high values of *n* and indicates the transition of electrons to the conduction bands. The σ/τ plots of SmMnO_3_ and EuMnO_3_ reach their maximum value at *T* = 1800 K, where EuMnO_3_ and GGA + *U* give higher values. From the curves of power factor (*S*^2^σ/τ) for the electron charge carriers shown in Figure 5d, we can see that they are also directly proportional to *T* and increase rapidly above *T* = 150 K (SmMnO_3_) and 300 K (EuMnO_3_), which is accompanied by semilinear rise up to *T* = 1600 K. Thus, the values of *S*^2^σ/τ indicate that these two perovskites have good thermoelectric properties with strong thermoelectric efficiency at a higher *T*.

Moreover, the thermoelectric efficiency of SmMnO_3_ and EuMnO_3_ can be judged by computing their thermoelectric figure of merit (*ZT*) using the relation
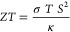
22

This clarifies the dependence of thermoelectric efficiency and *ZT* value on σ, *T*, *S*, and thermal conductivity (κ). The results of computed *ZT*, which correspond to the hole and electron charge carriers, for the two perovskites are shown in Table 6 and designed in Figure 6a, using GGA and GGA + *U*. From the two plots of electron charge carrier, we found that the GGA + *U* values of *ZT* at *S*_max_ are approximately equivalent with (*ZT* = 0.9999) at (*T* = 150 K). Therefore, it can be concluded that the two perovskites SmMnO_3_ and EuMnO_3_ with a high thermoelectric *ZT* are appropriate materials for thermoelectric applications and can be utilized in cooling systems. Furthermore, the total thermal conductivity (κ) is equal to the sum of lattice part (κ_L_) and electronic contributions (κ_E_)^37,40^
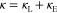
23

Figure 6b shows the change of κ_E_ and κ_L_ relative to τ (κ/τ), as a function of *T* for SmMnO_3_ and EuMnO_3_, using GGA and GGA + *U*. It can be observed that κ_*E*/τ_ increases with *T*, where this tendency is similar for these two HM perovskites. Finally, the specific heat capacity (*C*_V_) for these perovskites is computed and their variations with *T* are illustrated in Figure 6c. It can be seen that the value of *C*_V_ remains at zero (*C*_V_ = 0) up to (*T* = 150 K) for perovskite SmMnO_3_ and up to (*T* = 400 K) for perovskite EuMnO_3_, and above these points, the value of *C*_V_ increases rapidly (*C*_V_ > 0) with increasing *T*.

### 3.8 Optoelectronic Properties

Useful information for the electronic polarizability of the electrons in two cubic crystal structures of perovskites under study SmMnO_3_ and EuMnO_3_ can be obtained principally by computing the optical dielectric function ε(ω). It describes the optoelectronic interaction between applied electromagnetic radiation and the crystal structures. The function ε(ω) depends on the photon energy (ω) and can be defined as a complex sum of two parts: real part ε_1_(ω) and imaginary part ε_2_(ω)
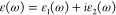
24

The Kramers–Kronig transformations (KKTs)^38,39^ give these parts as
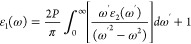
25and
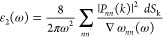
26where *P* is the principle part of integral, ω_*nn*_′ is the energy difference between ω and ω′ energies of the electronic states *n* and *n*′, respectively, *P*_*nn*_′(*k*) is the electric-dipole matrix element between *n* and *n*′ states, and *dS*_k_ is the energy surface. The two parts ε_1_(ω) and ε_2_(ω) in eqs 24–26 represent the measurements of dispersion and absorption of the electromagnetic radiation by the crystals.^38,47^

Based on ε(ω) and its parts, ε_1_(ω) and ε_2_(ω), we can acquire various optical parameters like the refractive index *N*(ω) as a sum of real part *n*(ω) and imaginary part *k*(ω) via
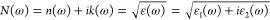
27where the real part is the refractive index *n*(ω) is
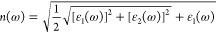
28

And the imaginary part refers to extinctive index *k*(ω)
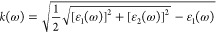
29

The absorption coefficient *α*(ω) can be computed by
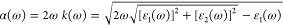
30

Very weak optical absorbing with lesser value of α(ω) indicates that *k*(ω) is also very small; this case provides the values
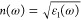
31
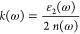
32

Also, there are other important optical parameters, i.e., reflection coefficient *R*(ω), that characterizes the part of energy reflected from the interface of the crystals. Its value is computed by
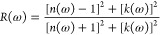
33or by using the value of the optical dielectric function ε(ω)
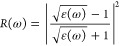
34

The optical conductivity σ(ω) is
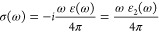
35

Furthermore, due to the incident of electromagnetic waves on the crystal structures, they cause inelastic scattering of their valence electrons, which results in a loss of electron energy. It is described via the electron energy loss function *L*(ω) and terms as imaginary part (Im) of inverse ε(ω)
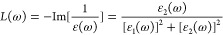
36

**Figure 7:**
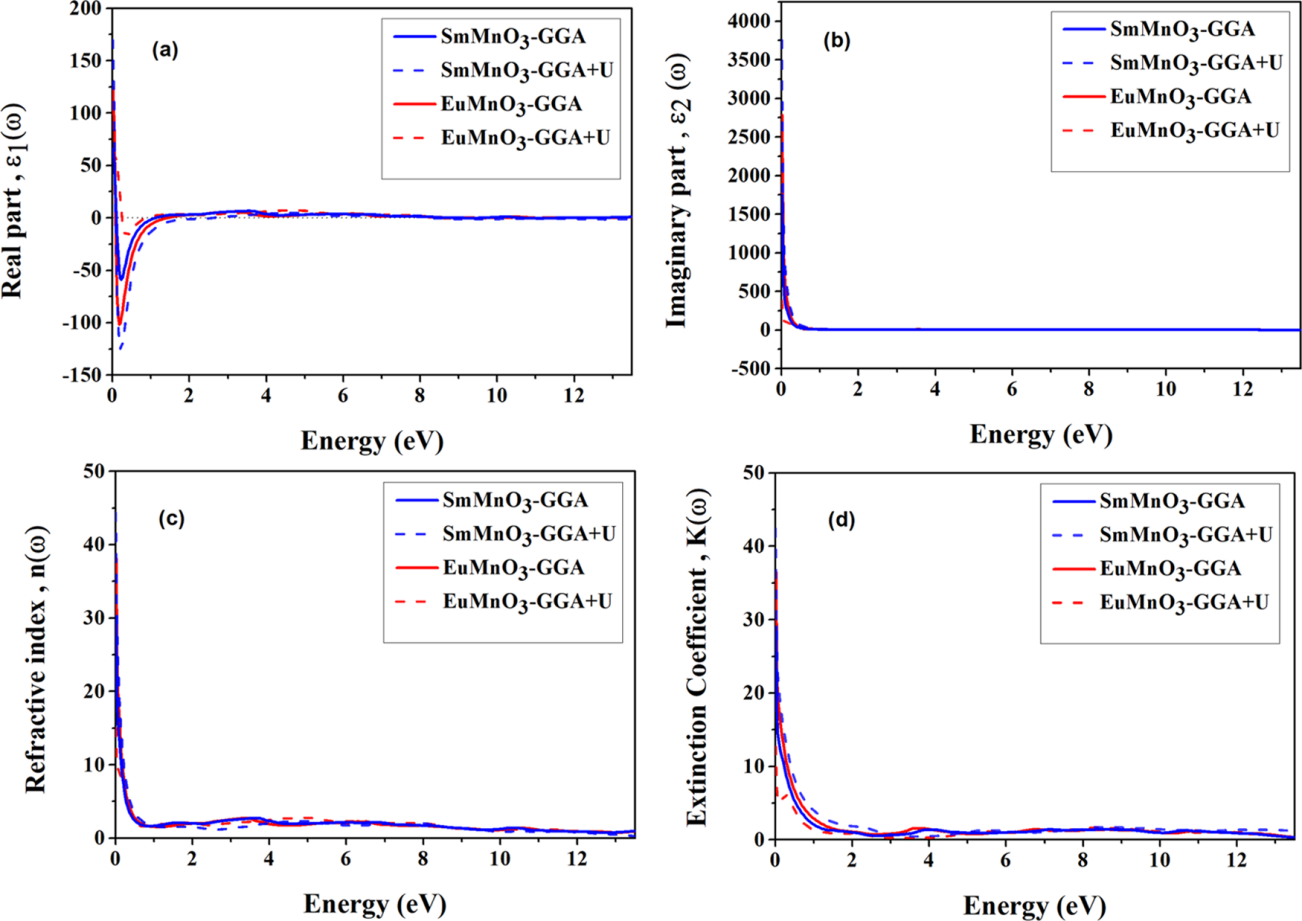
Computed optical properties of perovskites SmMnO_3_ and EuMnO_3_; (a) real part ε_1_(ω), (b) imaginary part ε_2_(ω) of dielectric function ε(ω), (c) refractive index *n*(ω), and (d) extinction coefficient *k*(ω), as a function of photon energy (ω), using GGA and GGA + *U*.

**Figure 8:**
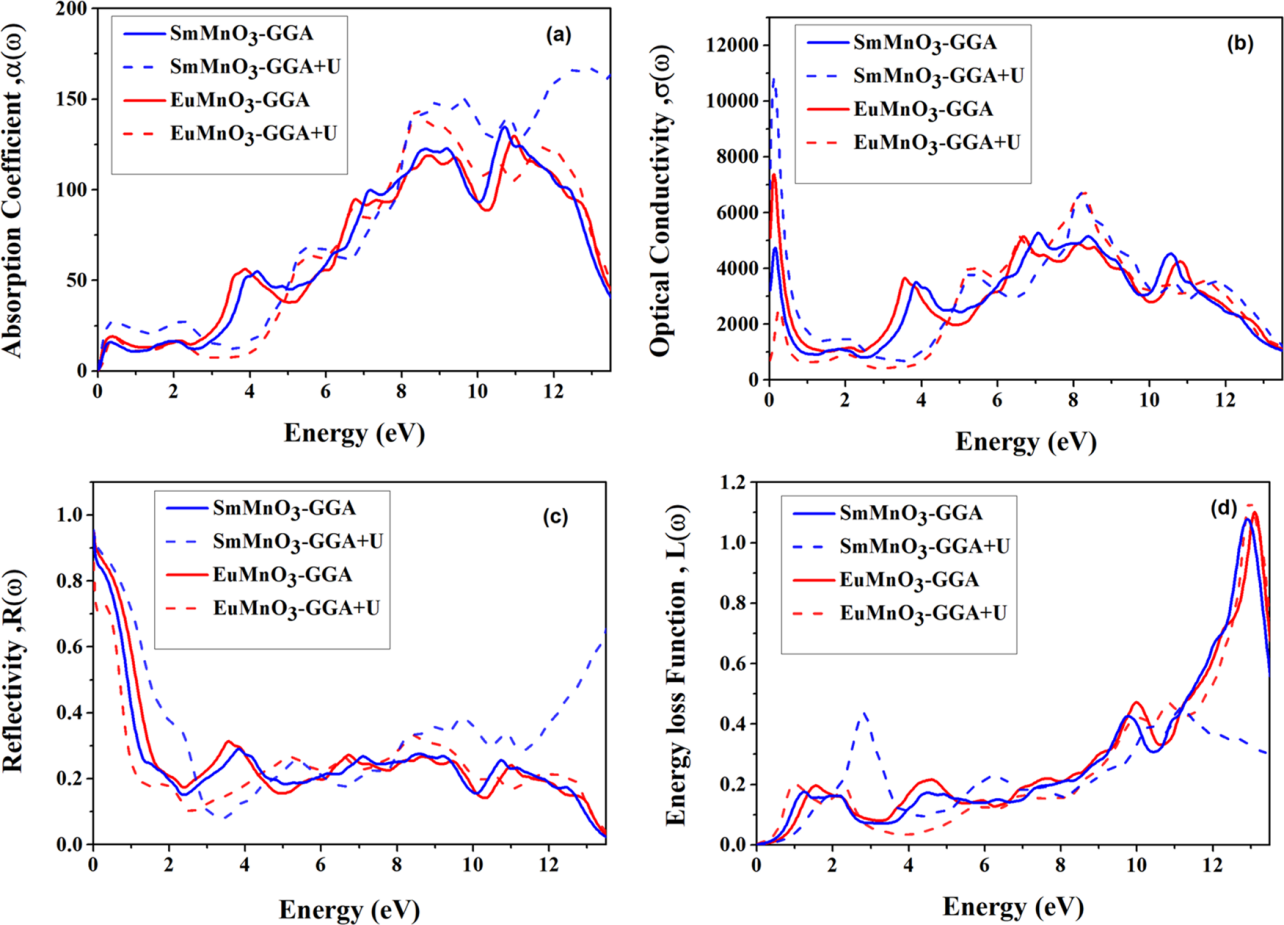
Computed optical properties of perovskites SmMnO_3_ and EuMnO_3_: (a) absorption coefficient α(ω), (b) optical conductivity σ(ω), (c) reflectivity *R*(ω), and (d) energy loss function *L*(ω), as a function of photon energy (ω), using GGA and GGA + *U*.

All of the above optical parameters are computed using GGA and GGA + *U* methods and shown in Figures 7 and 8. Since ε(ω) is an optical energy tensor that has three components along the directions *x*, *y*, and *z* for the cubic crystal structure of perovskites SmMnO_3_ and EuMnO_3_, it is enough to study the different optical parameters only along the *x* direction. Figure 7a,b corresponds to the spectra of ε_1_(ω) and ε_2_(ω) for SmMnO_3_ and EuMnO_3_, respectively, computed using the Korringa–Kohn–Rostoker (KKR) method.^19^ From Figure 7a, after ε_1_(0), the ε_1_(ω) spectra start to decrease sharply and reach negative values, then ε_1_(ω) approaches zero at high energies. The ε_2_(ω) spectra in Figure 7b demonstrate the optical ability of crystal structures to absorb the electromagnetic waves at energy ranges, where these spectra measure the total transport of electrons from the occupied valence band states to the unoccupied states in the band structures of conduction bands. Similarly, the ε_2_(ω) spectra confirm the metallic nature of two crystal structures of perovskites SmMnO_3_ and EuMnO_3_. This property is attributed mainly to high ε_2_(ω), which characterizes the metallic perovskites at zero frequency (ω = 0.0). Thus, both ε_1_(ω) and ε_2_(ω) indicate the HM nature of these perovskites within GGA and GGA + *U*.

The computed refractive index *n*(ω) and extinction coefficient *k*(ω) using GGA and GGA + *U* for perovskites SmMnO_3_ and EuMnO_3_ are shown in Figure 7c,d, respectively. Here, *n*(ω) evaluates how much the waves are refracted when entering the crystal structures and has a nature equivalent to ε_1_(ω) spectra. At low-energy regions, the *n*(ω) spectra in two crystals (Figure 7c) decrease rapidly from their maximum value to a point with energy of about 1.50 eV and then become semiflat curves at high-energy ranges. Similarly, the two *k*(ω) spectra (Figure 7d) show maximum value in the near-infrared range and then decrease up to a point with 3.0 eV. As seen in Table 7, the computed *k*_max_(ω) look similar and emerge in the energy range 0.0–3.0 eV.

**Table 7:** Computed Optical Properties of Perovskites SmMnO_3_ and EuMnO_3_

	SmMnO_3_		EuMnO_3_	
optoelectronic parameters	GGA	GGA + *U*	GGA	GGA + *U*
maximum extinction coefficient; *k*_max_ (ω)	29.069	42.341	36.161	12.441
optical conductivity; σ_max_ (ω) (Ω^–1^·cm^–1^)	5272.7 (at *E* = 7.06 eV)	10833.5 (at *E* = 0.12 eV)	6789.5 (at *E* = 8.26 eV)	7380.1 (at *E* = 0.13 eV)
static optical reflectivity; *R*(0)	0.8490	0.8570	0.8480	0.8580

Furthermore, the ε_1_(ω) and ε_2_(ω) parts of ε(ω) are utilized to compute the other optical parameters of two perovskites, along the *xx*-direction, including the absorption coefficient α(ω), optical conductivity σ(ω), reflectivity *R*(ω), and the energy loss function *L*(ω) using GGA and GGA + *U*, as shown in Figure 8. The absorption coefficient α(ω) describes the amount of energy required for interband transfer in crystal structures, where the α(ω) spectra show a number of peaks that can be illuminated by the interband transitions via the results of band structures. It is evident from Figure 8a that the α(ω) spectra of two perovskites SmMnO_3_ and EuMnO_3_ start at zero energy (*E* = 0.0 eV) and show high values at two different energy ranges (*E* = 8.0–10.0 eV) and (*E* = 11.0–13.0 eV) with small differences appear between the GGA and GGA + *U* spectra. In Figure 8b, we display the spectra of optical conductivity σ(ω), which show that the σ(ω) of SmMnO_3_ and EuMnO_3_ exhibit a metallic property since their photoconductivity begins at (*E* = 0.0 eV). The σ(ω) spectra have a range that encloses some peaks related to the bulk plasmon excitations induced by the electrons transferring from the occupied states in valence bands to unoccupied states in the conduction bands. The maximum optical conductivity σ_max_(ω) appears as a peak observed at different energy positions in the σ(ω) spectra (Table 7), where GGA + *U* spectra give values higher than those in GGA spectra.

Figure 8c shows the reflectivity *R*(ω) spectra, which are ascribed to the contributions of O^2–^-2p electrons in the valence bands and Mn^3+^-3d electrons in the conduction bands of SmMnO_3_ and EuMnO_3_. The static values of reflectivity *R*(0) for these two perovskites are computed using GGA and GGA + *U* and found to be about 85 and 86%, respectively (Table 7). Then, the *R*(ω) spectra of these compounds stay low until 1.90 and 1.60 eV for the GGA and till 3.50 and 2.50 eV for the GGA + *U*, respectively, which are consistent with the values of *E*_g_ (Table 5) obtained from GGA and GGA + *U* band structures and TDOSs, where the larger values of *E*_g_ obtained by the GGA + *U* method indicate a lower *R*(ω) value in the low-energy regions (0.0–4.0 eV) compared with those given by the GGA method. Finally, the computed spectra of energy loss function *L*(ω) that describes the fast electrons moving through the crystal structures are shown in Figure 8c. We can see that *L*(ω) spectra (Figure 8d) increase and an obvious peak arises due to the bulk plasmonic excitation at certain photon energy with a bulk plasma frequency (ω_p_).^32^ These ω_p_ situate at a high-energy range (*E* = 12.0–14.0 eV) in the SmMnO_3_ and EuMnO_3_ spectra; this energy corresponds with the rapid decrease in *R*(ω) spectra. The peaks’ location in the *L*(ω) spectra explains the transfer point from the metal to dielectric properties, where the crystals show dielectric properties, and beneath this point, they act as a metal.^5,32^

## Summary and Conclusions

In the present study, we reported the DFT computations of structural, elastic, thermal, optoelectronic, magnetic, mechanical, and thermoelectronic properties of two related manganite perovskites SmMnO_3_ and EuMnO_3_ using FP-LAPW skill. Their exchange–correlation potential has been treated via application of the Perdew, Burke, and Ernzerhof version of generalized gradient approximation (PBE-GGA) plus its corresponding Hubbard method (GGA + *U*). Initial results of FM structural optimization confirm the cubic symmetry (*Pm*3̅*m*) with minimum ground-state energy and equilibrium lattice constants (*a*_0_ ∼ 3.760–3.860 Å). The GGA and GGA + *U* computations of spin-polarized distributions of band structures and the partial and total density of states (DOS) distributions effectively predicted ferromagnetic (FM) plus half-metallic (HM) properties in two perovskites. Besides the effect of site replacement (RE^3+^ = Sm^3+^, Eu^3+^), the organized insertion of *U* energy within GGA + *U* computations has a major effect on all physical properties of SmMnO_3_ and EuMnO_3_, where GGA + *U* gives appropriate results. The distributions of electronic charge densities on the (110) plane confirmed a mix of ionic bonds (Sm^3+^/Eu^3+^–O^2–^) and covalent bonds (O^2–^–Mn^3+^–O^2–^), stabilized in an FM long-range exchange interaction (−Mn^3+^–O^2–^–Mn^4+^−). The obtained results of thermoelectronic properties for SmMnO_3_ and EuMnO_3_ show remarkable thermoelectronic responses with high electrical conductivity (σ/τ) at a high range of temperature (*T* = 1600 −1800 K) and negative value of maximum Seebeck coefficient (*S*_max_ = −2550 to −2720 μν/K). The predicted features help us to better realize these perovskites to play their best role as anode devices for solar fuel cells even at a high *T*. The present study will help to know the essential properties for the beneficial use in the high performance of solar cell materials. Crystal structures of both SmMnO_3_ and EuMnO_3_ showed that high dielectric constants, ε_1_(ω) and ε_2_(ω), and larger reflectivity *R*(ω) make these crystals appropriate materials for many spintronics and optoelectronic applications. The presence of low reflectivity *R*(ω) and electron energy loss function *L*(ω) at high energies advocates these materials favorable for optoelectronic device construction even at a higher range of electromagnetic wave energies. As a final remark, the existence of differences in the outcome physical properties of two perovskites is attributed mainly to the chemical nature of rare-earth atom occupying the RE^3+^ site plus the effect of utilizing GGA and GGA + *U* methods.

## Supporting Information Available

Crystal structure of perovskites SmMnO_3_ and EuMnO_3_; structural optimization of perovskite SmMnO_3_ in NM, FM, and AFM states using GGA and GGA + *U* methods; structural optimization of perovskite EuMnO_3_ in NM, FM, and AFM states using GGA and GGA + *U* methods; computed charge density of perovskites SmMnO_3_ and EuMnO_3_ using GGA and GGA + *U* methods; and computed structural properties of perovskites SmMnO_3_ and EuMnO_3_ in NM, FM, and AFM states (PDF)
