# Supplementary material for: Consequences of Tuning Rare-Earth RE3+-Site and Exchange–Correlation Energy U on the Optoelectronic, Mechanical, and Thermoelectronic Properties of Cubic Manganite Perovskites REMnO3 for Spintronics and Optoelectronics Applications
Source: ACS Omega. 2022 Aug 2;7(32):27903–17. doi: 10.1021/acsomega.2c01511 (PMC9386702; doi:10.1021/acsomega.2c01511)
Supplement: Supplementary file 1 — ao2c01511_si_001.pdf [file ao2c01511_si_001.pdf]

Supporting Information (SI)

Consequences of tuning rare-earth RE^{3+}-site and exchange-correlation energy U on the optoelectronic, mechanical and thermoelectronic properties of cubic manganite perovskites $REMnO_3$ for spintronics and optoelectronics applications

M. Musa Saad H.-E.[1,*], B. O. Alsobhi[2]

[1] Department of Physics, College of Science and Arts in Al-Muthnib, Qassim University, Saudi Arabia

[2] Physics Department, Faculty of Science, Taibah University, Al-Madinah al-Munawarah, Saudi Arabia

[*] Corresponding Author: Email: 141261@qu.edu.sa; Mob: +966509353808; ORCID: 0000-0002-6338-0915

Caption of supporting information

Figure S1. The crystal structure of perovskites $SmMnO_3$ and $EuMnO_3$

Figure S2. The structural optimization of perovskite $SmMnO_3$ in (a) NM, (b) FM and (c) AFM states using GGA method.

Figure S3. The structural optimization of perovskite $SmMnO_3$ in (a) NM, (b) FM and (c) AFM states using GGA+U method.

Figure S4. The structural optimization of perovskite $EuMnO_3$ in (a) NM, (b) FM and (c) AFM states using GGA method.

Figure S5. The structural optimization of perovskite $EuMnO_3$ in (a) NM, (b) FM and (c) AFM states using GGA method.

Figure S6. The computed charge density of perovskites (a) $SmMnO_3$ and (b) $EuMnO_3$.

Table S1. The computed structural properties of perovskites $SmMnO_3$ and $EuMnO_3$ in NM, FM and AFM states

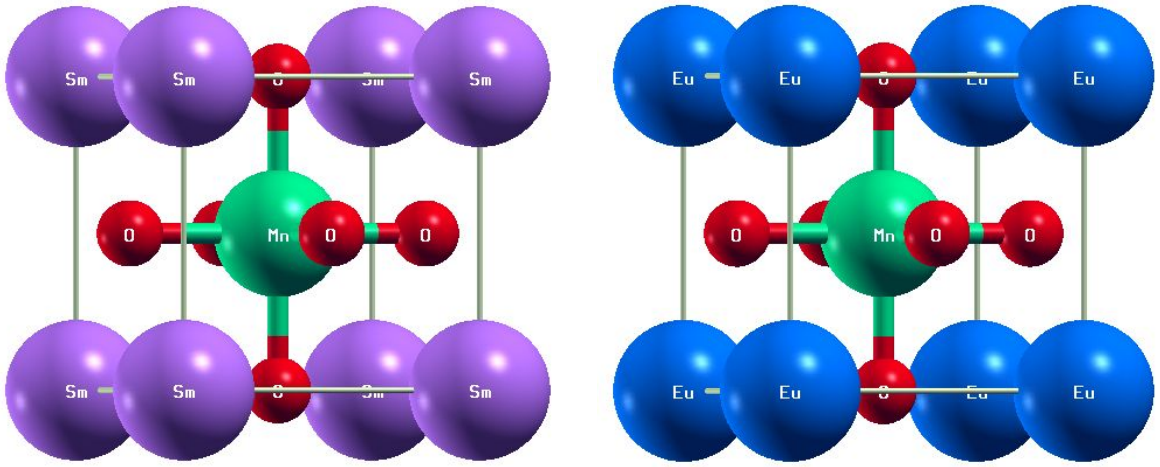

Figure S1. The crystal structure of perovskites $SmMnO_3$ and $EuMnO_3$

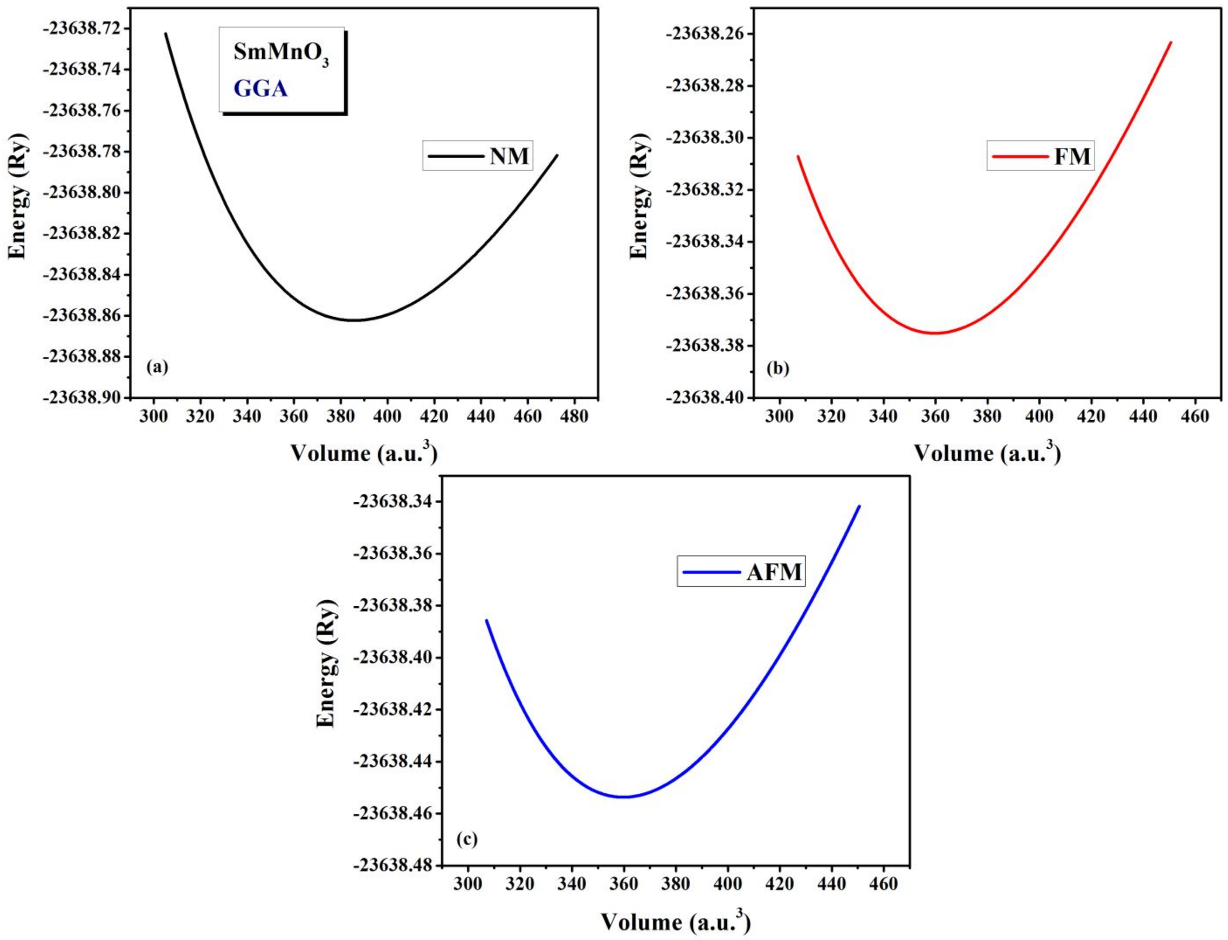

Figure S2. The structural optimization of perovskite $SmMnO_3$ in (a) NM, (b) FM and (c) AFM states using GGA method.

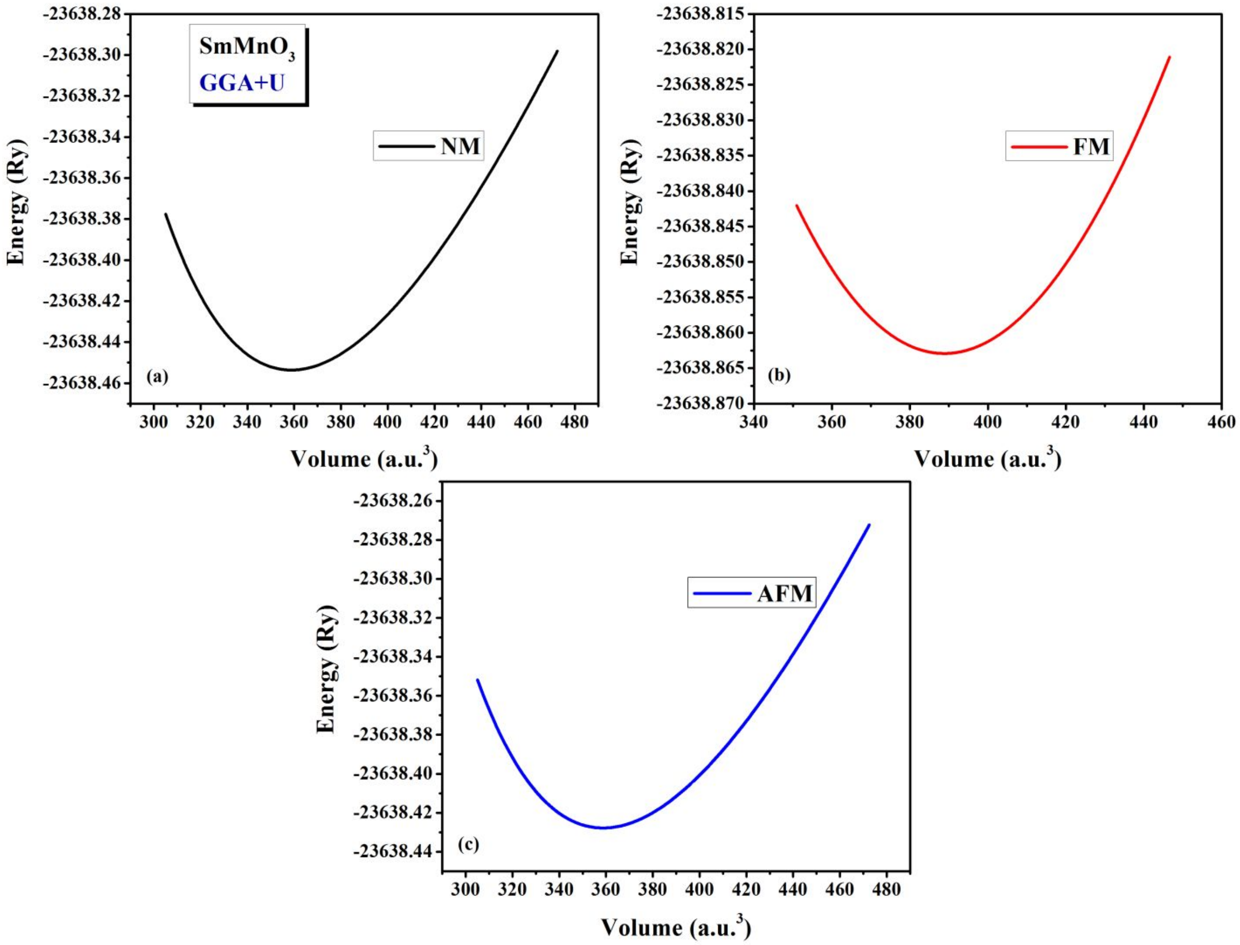

Figure S3. The structural optimization of perovskite $SmMnO_3$ in (a) NM, (b) FM and (c) AFM states using GGA+U method.

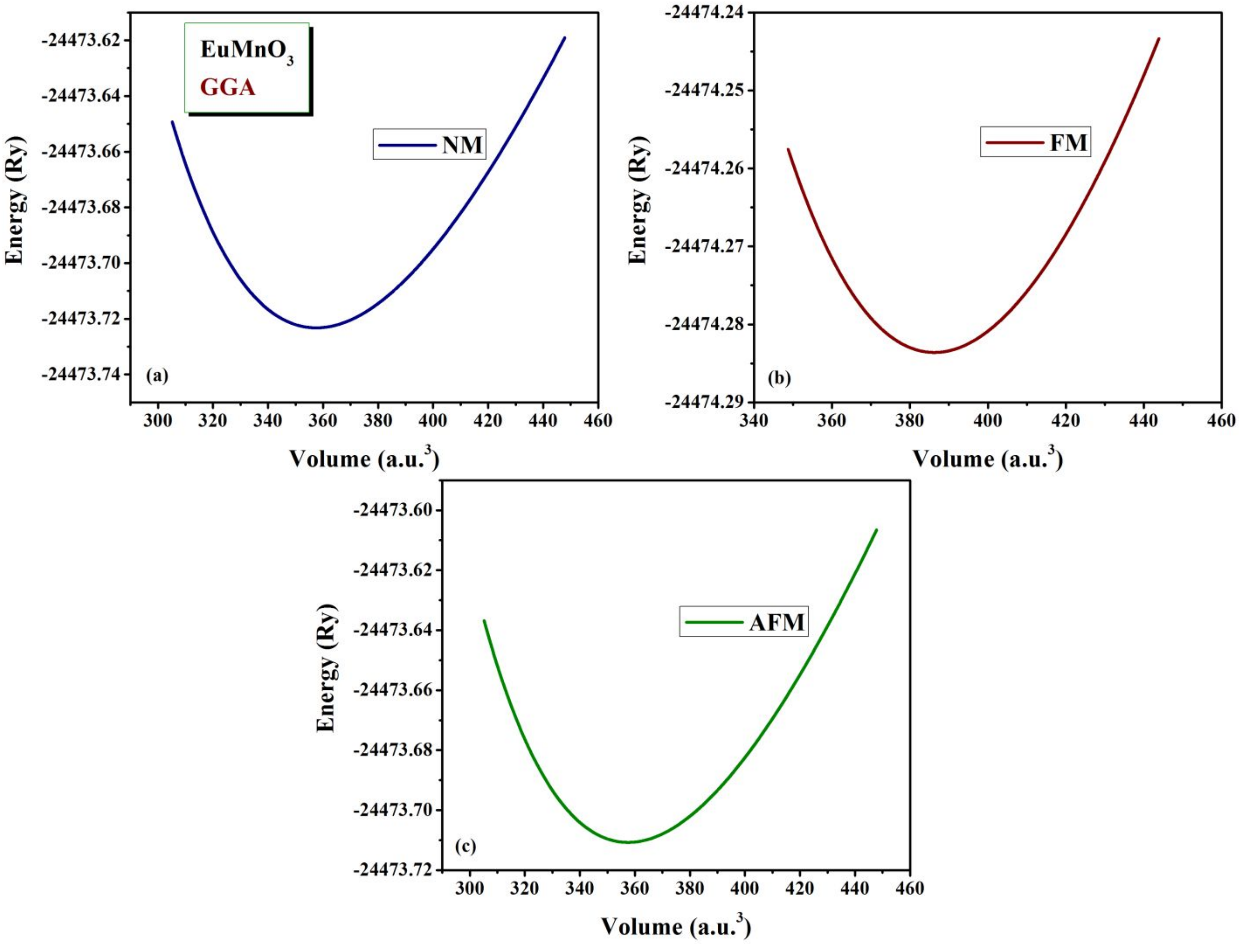

Figure S4. The structural optimization of perovskite $EuMnO_3$ in (a) NM, (b) FM and (c) AFM states using GGA method.

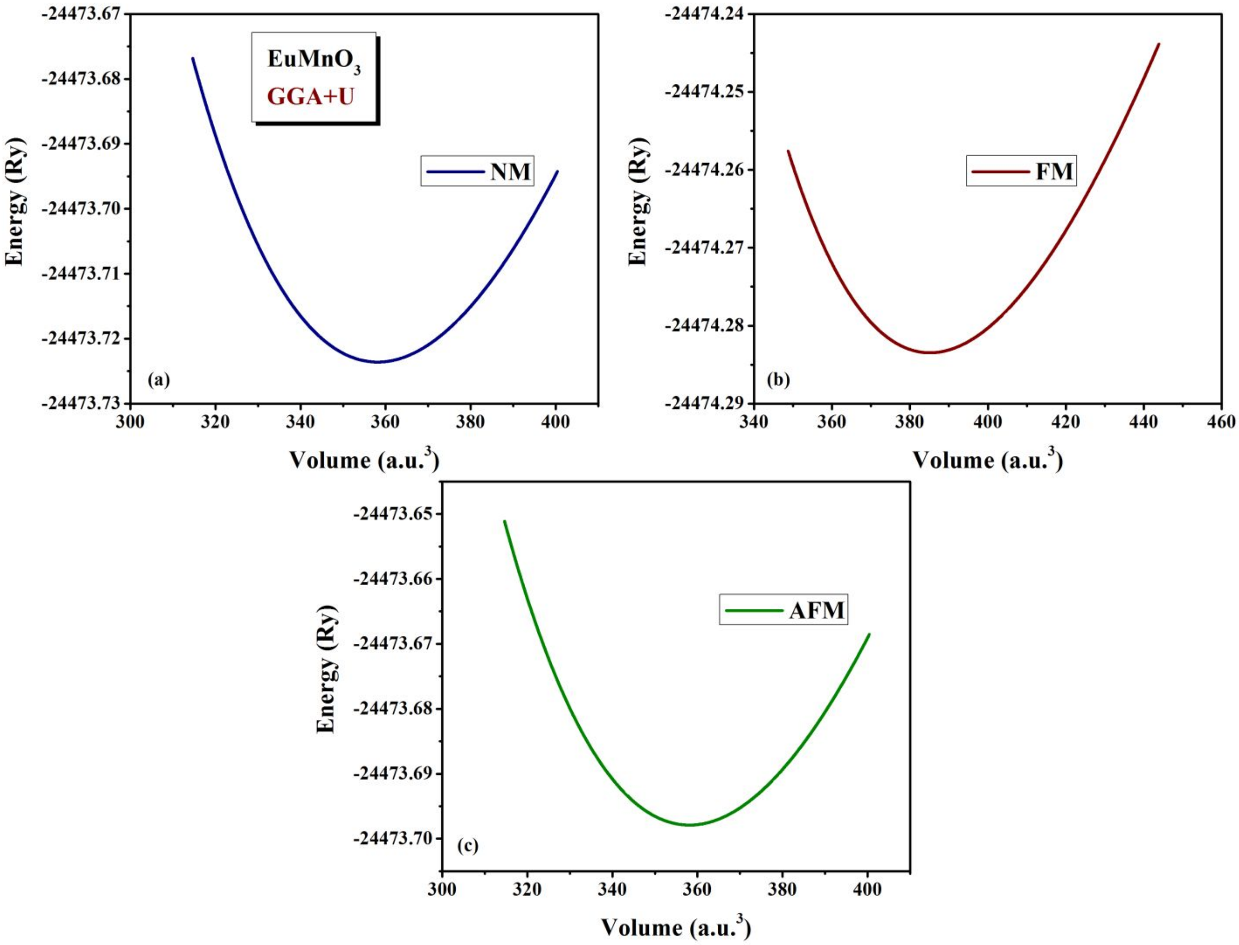

Figure S5. The structural optimization of perovskite $EuMnO_3$ in (a) NM, (b) FM and (c) AFM states using GGA method.

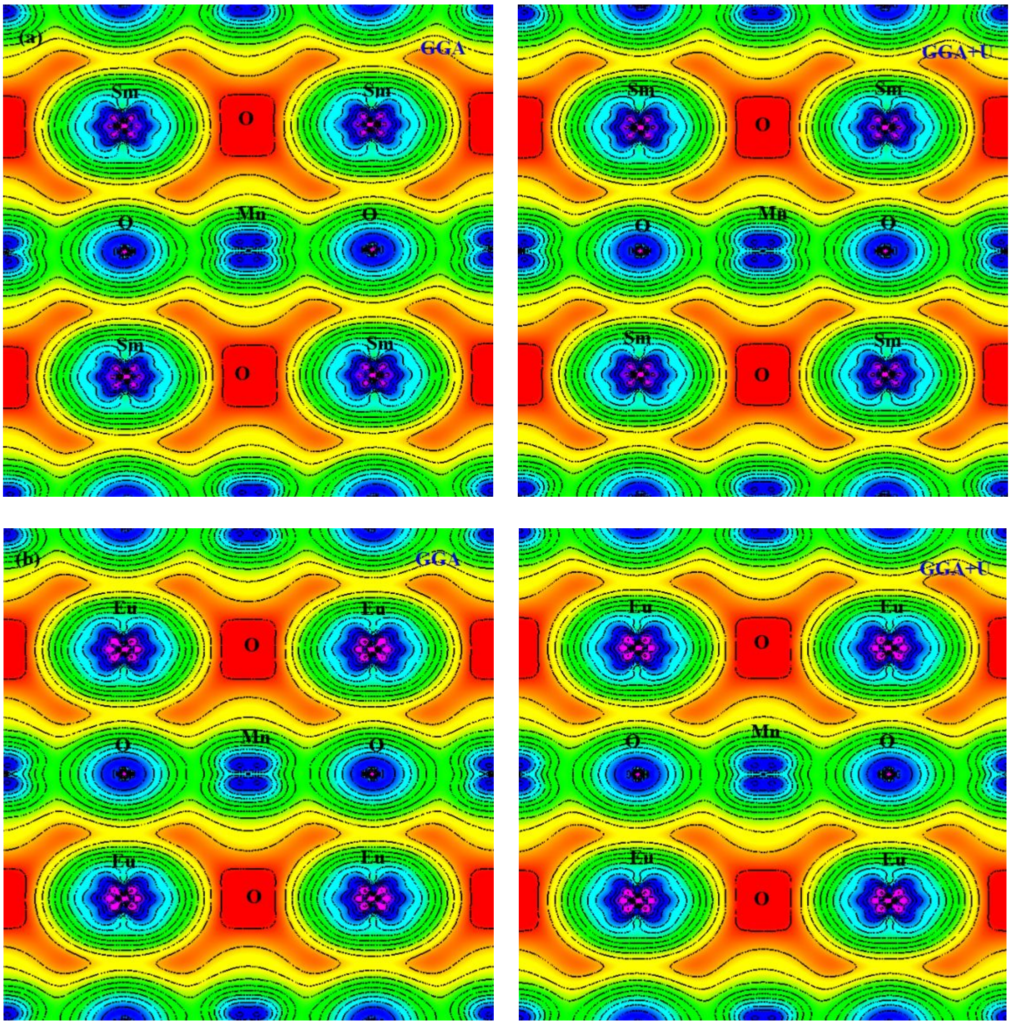

Figure S6. The computed charge density of perovskites (a) $SmMnO_3$ and (b) $EuMnO_3$.

Table S1. The computed structural properties of perovskites $SmMnO_3$ and $EuMnO_3$ in NM, FM and AFM states

$REMnO_3$		$SmMnO_3$		$EuMnO_3$	
Parameter	Phase	GGA	GGA+U	GGA	GGA+U
Lattice constant a_0 (Å)	NM	3.7635	3.7602	3.7558	3.7579
	FM	3.8520	3.8623	3.8539	3.8500
	AFM	3.7581	3.7662	3.7490	3.7577
Equilibrium volume V_0 (Å^3)	NM	53.306	53.166	52.980	53.065
	FM	57.154	57.617	57.239	57.068
	AFM	53.077	53.421	52.692	53.060
Bulk modulus $B_0(GPa)$	NM	201.55	204.46	204.77	208.00
	FM	165.75	156.22	175.03	176.38
	AFM	191.47	184.02	200.67	201.76
First-pressure derivative B_0'	NM	3.6770	4.4985	4.9347	4.0996
	FM	3.7685	4.8572	4.2987	5.7444
	AFM	3.6834	4.4592	4.8744	4.2754
Ground total energy E_0 (Ry)	NM	-23638.453676	-23638.453607	-24473.723237	-24473.723595
	FM	-23638.862296	-23638.862917	-24474.283599	-24474.283468
	AFM	-23638.453832	-23638.427881	-24473.710775	-24473.697128
Bond distance $RE - O$ (Å)	NM	2.6089	2.5568	2.6036	2.7415
Bond distance $Mn - O$ (Å)		1.8448	1.8079	1.8410	1.9389
Bond distance $RE - Mn$ (Å)		3.1953	3.1314	3.1887	3.3576
Bond distance $RE - O$ (Å)	FM	2.8855	2.8431	2.8372	2.8372
Bond distance $Mn - O$ (Å)		2.0404	2.0104	2.0062	2.0062
Bond distance $RE - Mn$ (Å)		3.5340	3.4821	3.4749	3.4749
	AFM	2.5829	2.5609	2.5776	2.7412
		1.8265	1.8108	1.8226	1.9387
		3.1634	3.1365	3.1569	3.3573
